# A web-based survey assessing perceived changes in diet, physical activity and sleeping behaviours in adults with type 1 and type 2 diabetes during the COVID-19 pandemic in the UK

**DOI:** 10.1136/bmjnph-2021-000391

**Published:** 2022-07-19

**Authors:** Charlotte Summers, Marjorie Lima Do Vale, Louise Haines, Sarah Armes, James Bradfield, Dominic Crocombe, Sumantra Ray

**Affiliations:** 1 DDM Health, Coventry, UK; 2 NNEdPro Global Centre for Nutrition and Health, Cambridge, UK; 3 Swiss Re Institute, Zurich, Switzerland; 4 School of Biomedical Sciences, Ulster University, Coleraine, UK

**Keywords:** COVID-19, diabetes mellitus, sleep, dietary patterns

## Abstract

**Background:**

The COVID-19 pandemic may have contributed to poorer self-management (ie, diet, physical activity and sleep) of diabetes mellitus (DM), which might predispose individuals to more severe COVID-19 outcomes.

**Objective:**

The first objective was to capture perceived changes in diet, physical activity and sleeping during the COVID-19 pandemic in adults with type 1 (T1DM) and type 2 diabetes mellitus (T2DM) in the UK. A second objective was to explore differences between individuals with DM compared with ‘no’ or ‘other’ health conditions.

**Methods:**

Participants aged >18 years were selected by convenience. Individuals subscribed to the Diabetes.co.uk community were sent a web-based survey including questions about demographics and health, followed by 5-point Likert-type scale questions relating to lifestyle-related behaviours during the COVID-19 pandemic. Individuals were grouped by diagnosis of DM, ‘other’ or ‘no’ health condition and responses were compared.

**Results:**

4764 individuals responded, with 2434 (51.3%) being female and 1550 (32.6%) aged 55–64 years. T2DM (2974; 62.7%), hypertension (2147; 45.2%) and T1DM (1299; 27.4%) were most frequently reported. Compared with T1DM, ‘no’ or ‘other’ health conditions, respondents with T2DM reported making a less conscious effort to get outside and exercise daily (p<0.001) and spending no time outdoors (p=0.001). Weight loss was more frequently reported in respondents with T2DM (p=0.005). More individuals with T2DM reported consuming convenience foods (p=0.012) and sugary foods (p=0.021), yet eating more fresh foods (p=0.001) and drinking less alcohol than normal (p<0.001). More individuals with T1DM and T2DM reported worse sleep quality (p=0.004).

**Conclusions:**

Our study highlighted important differences in lifestyle by individuals with T1DM, T2DM, other and no health conditions in relation to the COVID-19 pandemic. Establishing surveillance systems and conducting repeated assessments are required to analyse how the situation shifted over time and whether adverse collateral effects of the pandemic were sustained in those with chronic health conditions.

**WHAT IS ALREADY KNOWN ON THIS TOPIC**

- Disruptions to daily routines imposed by COVID-19 physical and social distancing measures have been associated with increased social isolation, anxiety and depressive symptoms, tendencies to consume unhealthier foods and being more sedentary.

**WHAT THIS STUDY ADDS**

- This study is one of the largest surveys in the UK reporting changes in lifestyle-related behaviours, including diet, physical activity and sleep among adults with type 1 and type 2 diabetes mellitus (T2DM) in the context of the first round of strict physical and social distancing measures imposed in 2020 in response to the COVID-19 pandemic.
- We found that individuals living with T2DM reported making a less effort to get outside and exercise (p<0.001), worse sleep quality (p=0.004), more frequent weight loss (p=0.005), greater consumption of convenience foods (p=0.012) and sugary foods (p=0.021), yet eating more fresh foods (p=0.001) and drinking less alcohol (p<0.001).
- Our findings highlighted that although the social and physical distancing measures imposed might have enabled some individuals to develop healthier habits, such as cooking more often and eating more fresh foods, it also likely had a negative impact on the habits and nutritional status of others.

**HOW THIS STUDY MIGHT AFFECT RESEARCH, PRACTICE AND/OR POLICY**

- Our research creates a better understanding of perceived lifestyle changes in individuals living with chronic health conditions, such as DM, during the early stages of the COVID-19 pandemic in the UK.
- Policymakers should closely monitor those who may be more vulnerable to the potential negative health impacts of socially restrictive policies in order to propose targeted protective measures in future public health responses.
- Researchers can contribute to this end by proactively studying the impacts of public health policies in a way that includes the real lived experiences of patients and the public.

## Background

Coronaviruses are a large family of viruses named for their appearance, which imitate the spikes on a crown.^1^ In December 2019, a novel coronavirus was discovered in China and subsequently named SARS-CoV-2. The disease caused by this virus became known as COVID-19.^2^ In March 2020, COVID-19 was further categorised as a pandemic by the WHO.^3^ Pandemics are declared when a new disease reaches worldwide spread, as has been observed with COVID-19.^4^ The COVID-19 pandemic has become a global burden that has resulted in millions of deaths worldwide. In the UK alone, more than 126 000 people died in the period of 1 year.^5^

To contain the spread of COVID-19 in the UK, measures to impose physical and social distancing, such as restrictions on movement, travel and social gatherings, closure of educational institutions and curfews, were widely implemented.^6^ These interventions likely impacted nutrition and lifestyle factors at individual, community, national and global levels.^7^ For instance, during the first month of lockdown, reports of increased social isolation, boredom and stress were published.^8^ Being confined in the home has been associated with tendencies to overeat^9^ and be more sedentary.^10–12^ Disruptions to daily routines imposed by physical and social distancing have also impacted anxiety and depressive symptoms.^13^ For example, during the first month of the UK containment measures, people reported increased anxiety as well as less motivation to eat healthily and reduced time exercising.^14^

Increased energy intake combined with decreased physical activity levels,^15^ as well as poor sleep^16^ and increased anxiety,^17^ is associated with poor metabolic outcomes, such as weight gain and increased insulin resistance. This is worrisome because metabolic diseases, such as obesity^18 19^ and diabetes mellitus (DM),^20^ put individuals at increased risk of more severe COVID-19 outcomes. Emerging evidence also suggests that physical and social distancing measures might disproportionately impact individuals with non-communicable diseases. For example, in the UK,^14^ the Netherlands^21^ and Germany,^22^ less healthy dietary and physical activity behaviours have been reported among individuals with a higher body mass index or those attending weight management services.

Changes in diet, physical activity, sleep and overall disease management have also been reported in individuals with diabetes. For instance, a survey conducted in Iran, Brazil and the USA during the COVID-19 lockdown reported increased food insecurity and a lack of spaces to exercise among individuals with type 1 diabetes mellitus (T1DM).^23^ In addition, inverse associations were found between access to healthy foods and HbA1c levels. Another survey conducted in the USA looking at the impact of COVID-19 on individuals with T1DM and type 2 diabetes mellitus (T2DM) found that almost half of the respondents reported negative effects on disease management, and about a quarter reported more frequent high glucose levels than before the pandemic. Differences between T1DM and T2DM participants were found in terms of glucose variability, with 22.5% of individuals with T1DM and 12.6% of those with T2DM reporting an increase in glucose variability.^24^ Potential differences between T1DM and T2DM were further suggested in a previous systematic review that appraised studies conducted in developed countries, including the UK. In this review, improved glycaemic control was reported among individuals with T1DM when COVID-19 lockdown measures were in place, yet this was not observed in individuals with T2DM, in whom glycaemic control deteriorated.^25^ Exploring the reasons for different outcomes between individuals with T1DM and T2DM can guide more tailored interventions that better support individuals’ needs in terms of diabetes management. For instance, a survey conducted in the UK reported that during the COVID-19 pandemic, individuals with T1DM and T2DM experienced decreased confidence in diabetes self-management, with 39.1% of those with T1DM reporting decreased confidence in terms of managing their mental well-being, 31.3% physical activity and 28.9% healthy eating pattern (vs 30.7%, 32.7% and 31.9% in those with T2DM, respectively). Individuals with T2DM also reported more difficulties obtaining information/advice and support,^26^ suggesting that this issue deserves further exploration.

Considering this, our study aimed to capture perceived changes in diet, physical activity and sleeping behaviours during the early stages of the COVID-19 pandemic in adults living with T1DM and T2DM in the UK. Our study also explored if there were differences between individuals living with T1DM and T2DM and individuals with ‘no’ or ‘other’ health conditions. Understanding these differences during the early stages of the COVID-19 pandemic can help relevant actors within the healthcare systems better comprehend how individuals within these groups responded to such an unprecedented scenario and in planning more tailored approaches to respond to their needs in the future.

## Methods

Between late March and June 2020, England experienced its first country-wide lockdown. Initially, all ‘non-essential’ high-street shops were closed. Individuals were told to stay at home, with permission to go only for ‘necessary’ purposes, such as purchasing food or seeking medical attention. The rules were gradually loosened between May and June 2020, with people allowed to leave their homes for outdoor pleasure (other than exercise) and gather outside in groups of up to six persons.

Most lockdown restrictions had been lifted by the time this survey was administered in July 2020. Many hospitality establishments were allowed to reopen, and gatherings of up to 30 individuals were permitted. However, the government nevertheless advised people to avoid gatherings larger than six and individuals were advised to follow the existing 2 m social distancing rule.

### Study design

This was a cross-sectional, web-based survey. Data collection occurred between 6 July 2020 and 31 August 2020.

### Recruitment and data collection

Convenience sampling was used to recruit participants. An email invitation, which included the link to complete the survey, was sent to 11 213 people aged ≥18 years who had voluntarily joined the online community Diabetes.co.uk and had previously consented to be contacted with research opportunities. All respondents were required to provide online informed consent by clicking on a required checkbox. Respondents who did not consent were excluded from the data analysis. The survey was administered through the Jisc Online Surveys software.

### Survey instrument

The survey instrument was developed by the research team. It comprised closed-ended questions organised across four sections, including (1) demographic characteristics, (2) health conditions, (3) COVID-19 symptoms and clinical diagnosis, and (4) lifestyle (physical activity, eating and sleeping) behaviours. Responses to lifestyle questions were assessed using a 5-point Likert scale. Participants were encouraged to complete all the questions.

One open-ended question collected information on participants’ opinions on how to end the COVID-19 situation, but responses will not be reported in this manuscript. The survey questionnaire is available in the online supplemental material.

10.1136/bmjnph-2021-000391.supp1 Supplementary data

### Statistical analysis

Demographic data were summarised using absolute and relative frequencies, as well as means and SDs. Categorical and ordinal variables used to assess lifestyle behaviours were described using absolute and relative frequencies. Considering the nature of the data and the fact that it did not satisfy the assumptions for the use of parametric tests, such as not being normally distributed and the notable differences in group sample sizes, non-parametric tests were used. Kruskal-Wallis tests followed by Dunn’s pairwise tests using the Bonferroni correction were carried out to assess statistical differences across groups (ie, T1DM, T2DM, ‘other’ or ‘no’ health condition). Missing data were subject to pairwise deletion for each analysis, which accounts for the variation in n. Bonferroni correction was applied to all Kruskal-Wallis tests to reduce the likelihood of false-positive results resulting from multiple comparisons, with statistical significance set at p<0.05 after the correction was applied. For the purpose of analysis, the T2DM group comprised participants reporting any of the following conditions: T2DM, pre-diabetes, metabolic syndrome or obesity. ‘Other health’ condition group reported anything other than the T1DM or T2DM. Analyses were performed using SPSS V.22.0 (SPSS).

## Results

Of the 11 213 people invited, 10 705 respondents started the survey (95.5% participation rate). About 4764 respondents completed the survey (42.5% response rate).

### Characteristics of participants

A little over half of respondents were female (2435; 51.3%), aged 55–64 years (1550; 32.6%), white (4434; 93.4%) and retired (2298; 48.7%). The most commonly reported health diagnoses were T2DM (2974; 59.5%), hypertension (2147; 42.9%), T1DM (1299; 26%), high cholesterol (1229; 24.6%) and arthritis (1002; 20.0%). Details of demographics are shown in table 1.

**Table 1:** Characteristics of respondents

Characteristics	T2DM	T1DM	Other health conditions	No health conditions	Total
	n (%)	n (%)	n (%)	n (%)	n (%)
Gender					
Male	1624 (52.6)	530 (41.4)	92 (35.5)	54 (46.2)	2287 (48.2)
Female	1446 (46.9)	746 (58.3)	165 (63.7)	62 (53.0)	2435 (51.3)
Prefer not to say	15 (0.5)	3 (0.2)	2 (0.8)	1 (0.9)	21 (0.4)
Age					
18–24	0 (0)	23 (1.8)	0 (0)	0 (0)	23 (0.5)
25–34	12 (0.4)	83 (6.5)	4 (1.5)	4 (4.4)	104 (2.2)
35–44	91 (2.9)	181 (14.1)	12 (4.6)	11 (12.2)	298 (6.3)
45–54	446 (14.4)	323 (25.2)	46 (17.6)	17 (18.8)	839 (17.6)
55–64	1020 (33.0)	404 (31.5)	80 (30.7)	34 (37.8)	1550 (32.6)
65–74	1193 (38.6)	227 (17.7)	91 (34.9)	19 (21.1)	1533 (32.2)
75 or older	331 (10.7)	42 (3.3)	28 (10.7)	5 (5.6)	410 (8.6)
Ethnicity					
Indian/Pakistani	45 (1.5)	12 (0.9)	4 (1.5)	5 (5.6)	69 (1.5)
Black/British African/Caribbean	31 (1.0)	6 (0.5)	6 (2.3)	2 (2.2)	46 (1)
Middle Eastern	2 (0.1)	2 (0.2)	1 (0.4)	0 (0)	5 (0.1)
Mixed groups	17 (0.6)	6 (0.5)	3 (1.2)	1 (1.1)	27 (0.6)
White	2890 (93.6)	1212 (94.8)	236 (90.8)	75 (83.3)	4434 (93.4)
Other	24 (0.8)	12 (0.9)	4 (1.5)	1 (1.1)	44 (0.9)
Chinese/Japanese/East Asian	4 (0.1)	2 (0.2)	0 (0)	1 (1.1)	7 (0.1)
I’d prefer not to say	73 (2.4)	27 (2.1)	6 (2.3)	5 (5.6)	113 (2.4)
Employment					
Full time	655 (20.9)	457 (35.0)	58 (21.9)	28 (30.8)	1205 (25.6)
Part-time	314 (10.0)	201 (15.4)	32 (12.1)	22 (24.2)	571 (12.1)
Retired	1728 (55.1)	387 (29.6)	136 (51.3)	31 (34.1)	2298 (48.7)
Student	12 (0.4)	22 (1.7)	4 (1.5)	0 (0)	39 (0.8)
Unemployed	271 (8.6)	158 (12.1)	26 (9.8)	5 (5.5)	464 (9.8)
Furloughed	109 (3.5)	67 (5.1)	6 (2.3)	4 (4.4)	188 (4)
Volunteering in my community (National Health Service (NHS), key services)	45 (1.4)	14 (1.1)	3 (1.1)	1 (1.1)	69 (1.5)
Number of comorbidities among respondents					
0	414 (13.4)	355 (27.6)	101 (38.7)	91 (100)	961 (20.2)
1	538 (17.4)	274 (21.3)	62 (23.8)	0 (0)	881 (18.5)
2	603 (19.5)	224 (17.4)	39 (14.9)	0 (0)	870 (18.3)
3	469 (15.1)	145 (11.3)	21 (8.0)	0 (0)	638 (13.4)
4	328 (10.6)	100 (7.8)	17 (6.5)	0 (0)	446 (9.4)
5	267 (8.6)	73 (5.7)	11 (4.2)	0 (0)	355 (7.5)
6+	479 (15.5)	113 (8.8)	10 (3.8)	0 (0)	613 (12.9)

T1DM, type 1 diabetes mellitus; T2DM, type 2 diabetes mellitus.

### COVID-19 symptoms and clinical diagnosis

A small proportion of respondents reported a clinical diagnosis of COVID-19 (115; 2.4%) or reported that a member of their household had been clinically diagnosed with COVID-19 (131; 2.8%). Of the 115 respondents who had been diagnosed with COVID-19, the majority (73; 63.5%) reported that their symptoms were severe or very severe.

### COVID-19 mitigation measures

The majority of respondents mentioned wearing a mask as a measure to reduce the spread of COVID-19 (940; 88.3%). Before the vaccines were approved and rolled out, when asked whether they would rather take a vaccine or use a digital app/service for monitoring the rate of transmission and spread of COVID-19, the majority preferred a vaccine (1348; 31.8%) with 606 (12.8%) preferring a digital app or service.

### Physical activity

**Figure 1:**
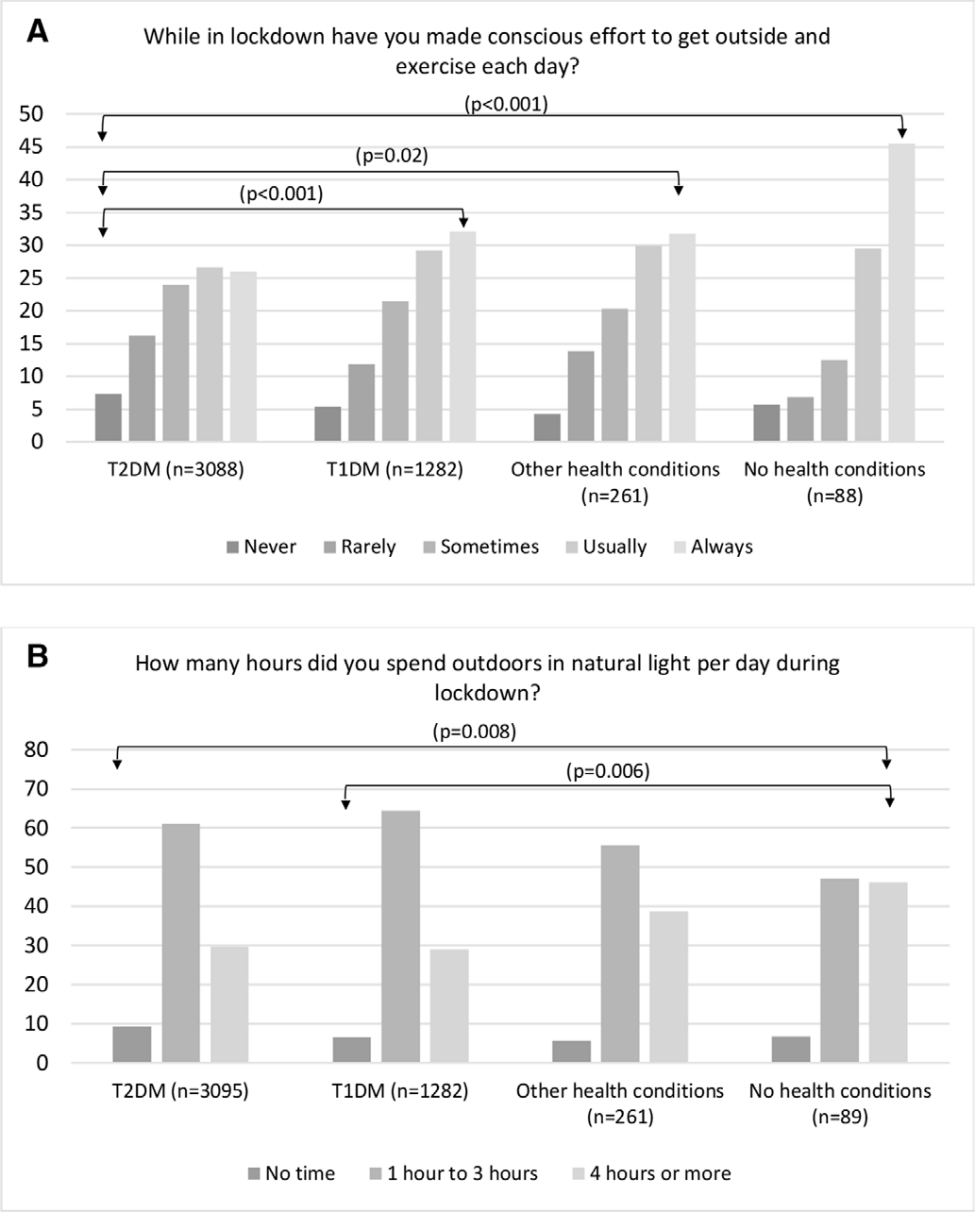
Physical activity and hours spent outdoors in the groups of type 2 diabetes mellitus (T2DM), type 1 diabetes mellitus (T1DM), other health conditions and no health conditions during lockdown. Arrows indicate statistically significant differences (p<0.05) between the two groups. The Kruskal-Wallis test and Dunn’s pairwise tests were carried out for the four pairs of groups, adjusted using the Bonferroni correction.

In total, 28.3% (1343) of respondents reported always making a conscious effort to get outside and exercise each day during the lockdown. Fewer individuals with T2DM made a conscious effort to get outside and exercise than those with T1DM (p<0.001), ‘other’ (p=0.020) or ‘no’ health condition (p≤0.001) (figure 1A). Compared with those with ‘no’ health conditions, individuals with T2DM (p=0.008) and T1DM (p=0.006) spent less time outdoors in natural sunlight overall (figure 1B).

### Diet

**Figure 2:**
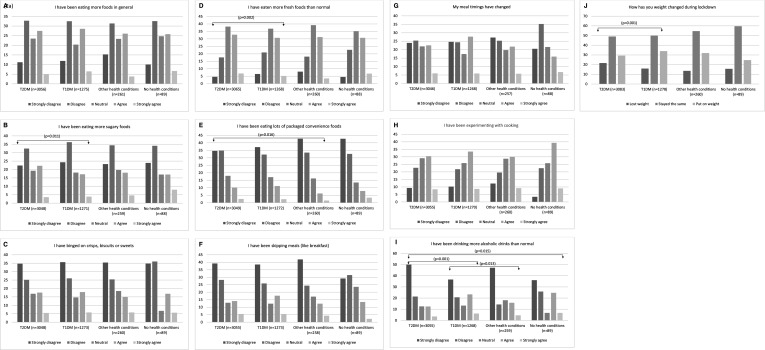
Changes in dietary patterns in the groups of type 2 diabetes mellitus (T2DM), type 1 diabetes mellitus (T1DM), other health conditions and no health as a result of lockdown. Arrows indicate statistically significant differences (p<0.05) between the two groups. The Kruskal-Wallis test and Dunn’s pairwise tests were carried out for the four pairs of groups, adjusted using the Bonferroni correction.

Around 20.3% of respondents strongly agreed or agreed that they had skipped meals, 29.6% had changed their meal timings, 33.1% ate more foods in general and 39.8% were experiencing more with cooking, with no significant differences observed across groups. Whereas more individuals from the T1DM group reported weight gain, the T2DM group more often reported weight loss (p<0.001) (figure 2J). In terms of specific food and drinks consumption, 23.1% strongly agreed or agreed that they had binged on crisps, biscuits or sweets, with no significant differences across groups. Compared with the T1DM group, more respondents from the T2DM group reported consuming more fresh (p=0.002) (figure 2D) but also more sugary (p=0.011) (figure 2B) foods. More respondents from the T2DM group also reported consuming more convenience foods when compared with the ‘other’ health condition group (p=0.016) (figure 2E). Compared with the T1DM (p<0.001) and ‘no’ health condition (p=0.015) groups, fewer individuals from the T2DM group reported consuming more alcohol. More participants from the T1DM group reported consuming more alcohol when compared with those with ‘other’ health conditions (figure 2I).

### Sleep

**Figure 3:**
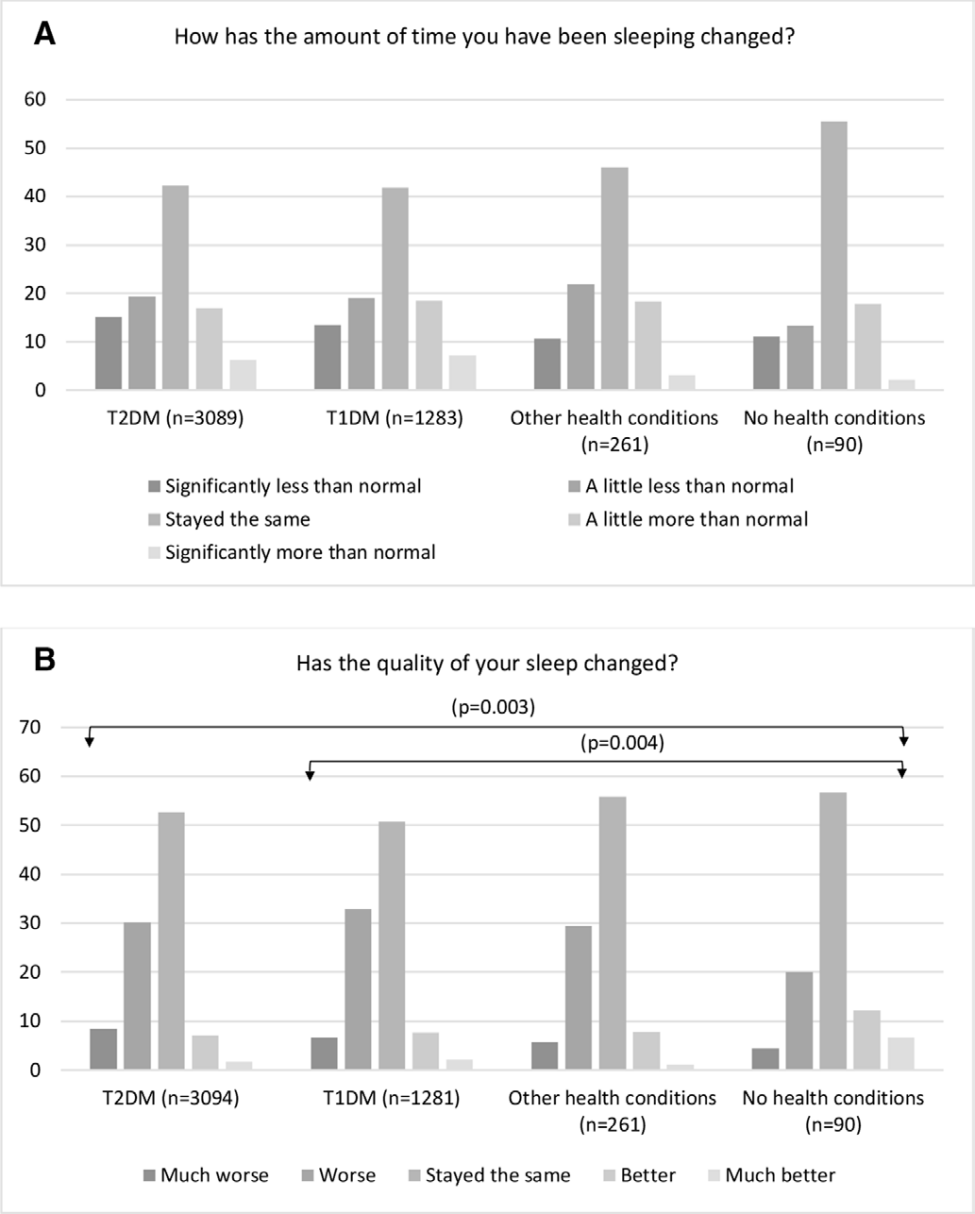
Perceived impact of COVID-19 on sleep amount and quality in the groups of type 2 diabetes mellitus (T2DM), type 1 diabetes mellitus (T1DM), other health conditions and no health conditions as a result of lockdown. Arrows indicate statistically significant differences (p<0.05) between the two groups. The Kruskal-Wallis test and Dunn’s pairwise tests were carried out for the four pairs of groups, adjusted using the Bonferroni correction.

Almost half of the respondents reported no changes in the amount of sleep (42.7%), with no significant differences across groups. More individuals with T1DM (p=0.004) or T2DM (p=0.003) reported worse sleep quality when compared with those with ‘no’ health condition (figure 3B).

## Discussion

Although not representative, this study is one of the largest surveys in the UK reporting changes in lifestyle-related behaviours, including diet, physical activity and sleep, among adults with T1DM and T2DM in the context of the first round of strict physical and social distancing measures imposed in 2020 in response to the COVID-19 pandemic. Our findings highlighted that although the social and physical distancing measures imposed might have enabled some individuals to develop healthier habits, such as cooking more often and eating more fresh foods, it also likely had a negative impact on the habits and nutritional status of others. For instance, fewer individuals with T2DM reported frequent outdoor exercise and spent fewer hours in natural sunlight compared with those with T1DM, ‘other’ or ‘no’ health condition, which is concerning considering that physical activity is beneficial to glycaemic control in patients with DM.^27^ Exercising outdoors was allowed during the first lockdown in the UK; however, individuals deemed ‘clinically extremely vulnerable’ due to increased risk of more severe COVID-19 outcomes were asked to stay at home almost all the time and avoid social contact with others.^28^ People with DM were not considered ‘clinically extremely vulnerable’ in UK government policy, but DM is known to increase an individual’s odds of poorer COVID-19 outcomes,^29^ and this widely reported knowledge may have persuaded people with DM to limit their outdoor activities. Although we did not ask respondents about indoor physical activity, other studies have corroborated reductions in overall physical activity during lockdown.^30^ An additional benefit of outdoor exercise is direct sunlight exposure to the skin, which is required for the synthesis of active vitamin D^31^; deficiency is strongly associated with COVID-19 severity and mortality.^32^

In our study, more respondents with T2DM increased the consumption of sugary and packaged convenience foods compared with those with T1DM. An increase in the consumption of sugary foods during lockdown among people with T2DM has also been reported elsewhere.^33^ Considering that people with T1DM are typically diagnosed earlier in life compared with those with T2DM, they might be more likely to manage their diets better. For instance, adherence to dietary recommendations for vegetable intake was reportedly higher in individuals with T1DM (44%) than in individuals with T2DM (36%).^34^ Another study in the context of the COVID-19 pandemic also reported inverse associations between time since diabetes diagnosis and eating distress.^25^

In this study, respondents with T2DM also reported worse sleep quality, trouble falling and/or remaining asleep, or oversleeping. This is supported by previous studies, suggesting that COVID-19 and lockdown impacted sleep, including increased sleep duration and delayed sleep onset.^35^ An Italian study found that 55% of participants reported poorer sleep quality during the COVID-19 lockdown, and poorer sleepers had higher levels of stress, anxiety and depression.^36^

Although the relationship between physical activity, diet and sleep has not been completely elucidated,^37 38^ they have been called the ‘big three’ factors for mental and physical health.^39^ This study showed that individuals with T2DM less often engaged in physical activity during the lockdown, along with increased intake of sugary, prepackaged convenience foods and worse sleep quality. This is similar to a previous study conducted with adults in Scotland, which reported positive correlations between changes in diet, physical activity and perceived sleep quality.^40^

The lockdown policies introduced by the UK government were an extreme measure deemed necessary by public health officials facing a novel pandemic. Measures that restrict the normal functioning of society in such a drastic are likely to impact individual lifestyle behaviours and health. It is essential that lessons are learnt from the experiences of people living through this period to better inform future policy decisions. The differences seen in responses to questions regarding food choice, eating behaviours, physical activity and sleep patterns highlight that considerations must be made for those subgroups of the population who may have been disproportionally affected by the pandemic and associated restrictive measures. A previous UK survey found that 41% of individuals with DM living alone did not receive outside support during the early phases of the pandemic, and 37% reported the quality of healthcare information, advice and support they received during this time to be poor.^28^ Policymakers would do well to include protective measures in future public health responses to support those who may be more vulnerable to the potential negative health impacts of socially restrictive policies. Researchers can contribute to this end by proactively studying the impacts of public health policies in a way that includes the real lived experiences of patients and the public.

### Strengths and limitations

This study is observational and therefore cannot necessarily prove causation. However, the value in reporting these findings is to highlight the real lived experience of this large patient and public group during an unprecedented public health crisis. Given the scale and impact of the pandemic and the lockdown policies, it is not unreasonable to attribute the lifestyle and behavioural changes reported by respondents specifically to the combined effects of lockdown policies and the pandemic itself. Indeed, some of the questions asked aimed to explore a temporal relationship, whereby the changes reported occurred after the onset of the pandemic and after the introduction of lockdowns. Regarding the differences in experiences reported between people with T1DM, T2DM, ‘other’ and ‘no’ health conditions, it is also plausible that those living with similar lifestyle-related health conditions exhibit similar lifestyle patterns that are distinct from others with different conditions or none.

This study has limitations. The data were collected in July 2020, a short period after the first round of severe restrictions in the UK was eased. As such, some responses are subject to each individual’s recall of changes made a few months prior (March to May 2020). Considering the timing of the study, we can only make inferences about the short-term impact of the lockdown on lifestyle behaviours. In addition, as this is an internet-based survey, there is a likelihood of selection bias towards those who usually engage with information technology, those who have access to the internet, are active online and who have previously signed up to be contacted with research opportunities. Our analysis did not account for differences in age, gender, socioeconomic status and multicomorbidities within the four groups. A higher percentage of individuals with T2DM were male, older and with more comorbidities. Furthermore, the T2DM group also included those with metabolic syndrome and pre-diabetic obesity whereby these two subgroups may have modulated the overall risk profile for T2DM in an apposing manner. It is also worth noting there was also a limited representation of individuals from different ethnic groups. Also, the majority of respondents in our survey were 65 years and older. The questionnaires used were not validated. Also, we did not ask about COVID-19 diagnostic tests used to confirm a COVID-19 infection, as this was not the focus of this research. We believe that individuals who reported having been infected with COVID-19 did so in line with National Health Service (NHS) messages available at that time, although there is a chance of misreporting.

## Conclusion

This study presented a cross-sectional snapshot highlighting important differences in lifestyle and diet-related behaviours by individuals with T1DM, T2DM, other and no health conditions in relation to the COVID-19 pandemic restrictions introduced by the UK government in March 2020. Establishing surveillance systems and conducting repeated assessments using validated tools are required next steps to analyse how the situation shifted over time and whether adverse collateral effects of the COVID-19 pandemic were sustained in those already living with chronic health conditions, such as DM. Such insights are crucial to understanding people’s lived experiences with DM and could be used to inform public health policy in the future.

## Data Availability

Data are available upon reasonable request.
