# Supplementary material for: A web-based survey assessing perceived changes in diet, physical activity and sleeping behaviours in adults with type 1 and type 2 diabetes during the COVID-19 pandemic in the UK
Source: BMJ Nutr Prev Health. 2022 Jul 19;5(2):137–44. doi: 10.1136/bmjnph-2021-000391 (PMC9813628; doi:10.1136/bmjnph-2021-000391)
Supplement: Supplementary data [file bmjnph-2021-000391supp001.pdf]

Before we start

In any crisis, the priorities are to solve the immediate problem and keep it from happening again. The COVID-19 pandemic is an example of this.

Whilst focus is on COVID-19 and adapting to new ways of living, we need to save lives and also improve the ways we respond to such outbreaks. Staying connected during COVID-19 has meant the use of digital services and channels that replace or augment traditional face-to-face practices.

In response to the pandemic, many organisations are creating services rapidly. Technological solutions to mitigate the COVID-19 crisis have been implemented in China and South Korea, for instance, where authorities avoided a complete closure of the country, succeeding to limit the spread of the virus. European governments are tempted to replicate such measures, with many believing this to be a quick fix solution. Such thinking raises a number of concerns, however, particularly with regards to European ethics, privacy and data protection legislation.1

This survey looks to understand your concerns when it comes to ethics of data privacy, the use of digital tools to track symptoms of COVID-19 and those diagnosed, and what you consider to be unmet needs and concerns.

First of all, we would like to know a little bit more about you.

1. What is your age?

- ○ 18 to 24
- ○ 25 to 34
- ○ 35 to 44
- ○ 45 to 54
- ○ 55 to 64
- ○ 65 to 74
- ○ 75 or older

2 What is your gender?

- ○ Male
- ○ Female
- ○ Other/prefer not to say

3. Have you had any of the symptoms of COVID-19?

- ☐ Continuous cough
- ☐ Fever
- ☐ Difficulty breathing
- ☐ Loss of smell/taste
- ☐ Other

4. If you selected Other, please specify:

____

5. Have you been clinically diagnosed with COVID-19?

- ○ Yes
- ○ No

5.a. How severe did you perceive your symptoms to be? (1: Not severe at all to 10: Very severe)

____

6. Has anyone in your household been tested and clinically diagnosed with COVID-19?

- ○ Yes
- ○ No

Health conditions

7. Please select any health conditions you have been diagnosed with:

- ☐ Allergies
- ☐ Alzheimer's disease
- ☐ Anxiety
- ☐ Arthritis
- ☐ Asthma
- ☐ Cancer
- ☐ Cardiovascular disease
- ☐ Chronic Kidney Disease (CKD)
- ☐ Chronic pain
- ☐ Coeliac disease
- ☐ COPD
- ☐ Crohn's Disease
- ☐ Dementia
- ☐ Depression
- ☐ Fatty liver disease
- ☐ Fibromyalgia
- ☐ Heart disease
- ☐ HIV
- ☐ High blood pressure / hypertension
- ☐ High cholesterol
- ☐ IBS
- ☐ Insomnia
- ☐ Metabolic syndrome
- ☐ Multiple sclerosis
- ☐ Nephropathy
- ☐ Neuropathy
- ☐ Obesity
- ☐ Other mental health
- ☐ Overactive bladder (OAB)
- ☐ Parkinson's Disease
- ☐ Prediabetes
- ☐ Polycystic Ovarian Syndrome (PCOS)
- ☐ Retinopathy
- ☐ Type 1 diabetes
- ☐ Type 2 diabetes
- ☐ Ulcerative Colitis
- ☐ Visual impairment
- ☐ I am not diagnosed with any condition by a medical professional
- ☐ Other

7.a. If you selected Other, please specify:

7.b. What was your latest HbA1c?

Diet and lifestyle

These next questions explore the perceived impact of COVID-19 on your overall health and lifestyle.

8. While in lockdown have you made a conscious effort to get outside and exercise each day?

- ○ Always
- ○ Usually
- ○ Sometimes
- ○ Rarely
- ○ Never

9. How has the amount of time you have been sleeping changed?

- ○ I have been sleeping significantly less than normal
- ○ I have been sleeping a little less than normal
- ○ Stayed the same
- ○ I have been sleeping a little more than normal
- ○ I have been sleeping significantly more than normal

10. Has the quality of your sleep changed?

- ○ Much better
- ○ Better
- ○ Stayed the same
- ○ Worse
- ○ Much worse

11. How have your dietary patterns changed as a result of lockdown?

Please don't select more than 1 answer(s) per row.

	Strongly disagree	Disagree	Neutral	Agree	Strongly agree
I have been eating more food in general	☐	☐	☐	☐	☐
I have been eating more sugary foods	☐	☐	☐	☐	☐
I have eaten more fresh foods than normal	☐	☐	☐	☐	☐
I have been experimenting with cooking	☐	☐	☐	☐	☐
I have been eating lots of packaged convenience foods	☐	☐	☐	☐	☐
I have been drinking more alcoholic drinks than normal	☐	☐	☐	☐	☐
I have been skipping meals (like breakfast)	☐	☐	☐	☐	☐
My meal timings have changed	☐	☐	☐	☐	☐
I have binged on foods such as crisps, biscuits, or sweets	☐	☐	☐	☐	☐

12. How has your weight changed during lockdown?

- ○ I have put on weight
- ○ I have stayed roughly the same weight
- ○ I have lost weight

13. How many hours did you spend outdoors in natural light per day during lockdown?

- ○ 0 (No time)
- ○ 1 hour
- ○ 2 hours
- ○ 3 hours
- ○ 4 hours
- ○ 5 hours
- ○ 6 hours
- ○ 7 hours
- ○ 8 hours
- ○ 9 hours
- ○ 10 hours
- ○ 11 hours
- ○ 12 hours or more

Your feelings

In these next questions, we would like to understand how you have been feeling.

14. Where are you currently residing?
Please enter the first half of your postcode

Your answer should be no more than 5 characters long.

☐

15. What is your current employment status?

- ☐ Full-time employment
- ☐ Part-time employment
- ☐ Retired
- ☐ Student
- ☐ Unemployment
- ☐ Furloughed
- ☐ Volunteering in my community (NHS, key services)

16. What is your race or ethnicity?

- ○ Indian/Pakistani
- ○ Black or British African/African/Caribbean
- ○ Middle Eastern
- ○ Mixed/Multiple ethnic groups
- ○ White
- ○ Other
- ○ Chinese/Japanese/Other East Asian
- ○ I'd prefer not to say

17. This question is about your feelings and thoughts during the last month. In each case, select how often you felt or thought a certain way. Please don't select more than 1 answer(s) per row.

	Never	Almost never	Sometimes	Fairly often	Often
In the last month, how often have you felt that you were unable to control the important things in your life?	☐	☐	☐	☐	☐
In the last month, how often have you felt confident about your ability to handle your personal problems?	☐	☐	☐	☐	☐
In the last month, how often have you felt that things were going your way?.	☐	☐	☐	☐	☐
In the last month, how often have you felt difficulties were piling up so high that you could not overcome them?	☐	☐	☐	☐	☐

18. Over the last 2 weeks, how often have you been bothered by the following problems?

Please don't select more than 1 answer(s) per row.

	Not at all	Several days	More than half the days	Nearly every day
Little interest or pleasure in doing things	☐	☐	☐	☐
Feeling down, depressed or hopeless	☐	☐	☐	☐
Trouble falling asleep, staying asleep, or sleeping too much	☐	☐	☐	☐
Feeling tired or having little energy	☐	☐	☐	☐
Poor appetite or overeating	☐	☐	☐	☐
Feeling bad about yourself - or that you're a failure or have let yourself or your family down	☐	☐	☐	☐
Trouble concentrating on things, such as reading the newspaper or watching television	☐	☐	☐	☐
Moving or speaking so slowly that other people could have noticed. Or, the opposite - being so fidgety or restless that you have been moving around a lot more than usual	☐	☐	☐	☐
Thoughts that you would be better off dead or of hurting yourself in some way	☐	☐	☐	☐

19. Over the last 2 weeks, how often have you been bothered by the following problems?

Please don't select more than 1 answer(s) per row.

	Not at all	Several days	More than half the days	Nearly every day
Feeling nervous, anxious or on edge	☐	☐	☐	☐
Not being able to stop or control worrying too much about different things	☐	☐	☐	☐
Trouble relaxing	☐	☐	☐	☐
Being so restless that it is hard to sit still Becoming easily annoyed or irritable	☐	☐	☐	☐
Feeling afraid as if something awful might happen	☐	☐	☐	☐

20. What would you like to see happen to improve the COVID-19 situation?

21. Pleas enter your email address if you would like to receive a copy of the report that is completed after the analysis of the outcomes:

Please enter a valid email address.
